# Classification of Chronic Obstructive Pulmonary Disease (COPD) Through Respiratory Pattern Analysis

**DOI:** 10.3390/diagnostics15030313

**Published:** 2025-01-29

**Authors:** Do-Kyeong Lee, Jae-Sung Choi, Seong-Jun Choi, Min-Hyung Choi, Min Hong

**Affiliations:** 1 Department of Software Convergence, Soonchunhyang University, Asan 31538, Republic of Korea; dooky606@daum.net; 2 Department of Internal Medicine, Cheonan Hospital, College of Medicine, Soonchunhyang University, Cheonan 31151, Republic of Korea; cjssch@schmc.ac.kr; 3 Department of Otolaryngology-Head and Neck Surgery, Cheonan Hospital, College of Medicine, Soonchunhyang University, Cheonan 31151, Republic of Korea; akas9238@hanmail.net; 4 Department of Computer Science, Saint Louis University, Louis MO 63103, USA; 5 Department of Computer Software Engineering, Soonchunhyang University, Asan 31538, Republic of Korea

**Keywords:** thermal camera, image preprocessing, COPD, non-contact diagnosis

## Abstract

**Background:** This study proposes a classification system for predicting chronic obstructive pulmonary disease (COPD) patients and non-patients based on image and text data. **Method:** This study measured the respiratory volume based on thermal images, stored the respiratory data, and derived features related to respiratory patterns, including the total respiratory volume, average distance between expirations, average distance between inspirations, and total respiratory rate. The data for each feature were stored in text format. The four features saved as text were scaled using Z-score normalization and expressed as scores through weighted summation. These scores were compared to a threshold based on the ROC curve values, classifying participants as patients if the score exceeded the threshold and as non-patients if it fell below. **Results:** The proposed method achieved an accuracy of 82.5%. To validate the proposed approach, precision, recall, and F1-score were utilized, confirming the high classification performance of the model. The results of this study demonstrate the potential for future applications in non-contact medical examinations and diagnoses of respiratory diseases.

## 1. Introduction

Chronic obstructive pulmonary disease (COPD) has shown an increasing prevalence worldwide due to population aging and has become a leading cause of morbidity and mortality among the elderly population [1,2,3]. Smoking and population aging have consistently been identified as major contributing factors to the development of COPD [4]. However, recent urbanization and lifestyle diversification have introduced various risk factors, including indoor air pollution, ambient air pollution, and exposure to occupational hazardous substances [5,6]. These factors complicate the prediction of the onset and progression of COPD, reducing our predictive power [7].

Therefore, accurate assessment of vital signs the early diagnosis of COPD plays a critical role in preventing the progression and worsening of the disease [8]. This is because COPD treatment primarily focuses on preventing the exacerbation of symptoms. The characteristic symptoms of COPD patients include coughing, sputum production, and shortness of breath. Due to irregular airflow, COPD often leads to exercise intolerance and various respiratory issues [9,10].

The current standard technique for pulmonary function testing (PFT) [11] involves the use of a spirometer, which is utilized for diagnosing COPD and conducting periodic pulmonary function assessments. However, the use of a spirometer requires a hospital visit, and measurements are performed using a mouthpiece to assess the airflow during inspiration and expiration.

Given the characteristics of COPD, which predominantly affects the aged population, visiting a hospital for pulmonary function measurement can pose regional constraints and incur financial costs [12,13]. Moreover, since the outbreak of COVID-19, the emphasis on minimizing contact and the growing interest in telemedicine have highlighted the need for non-contact diagnostic methods. Therefore, even the use of disinfected mouthpieces can cause discomfort for patients, and for COPD patients who are particularly vulnerable to respiratory diseases, it may pose significant risks. This necessitates the development of alternative methods for pulmonary function assessment [14,15,16].

Recent studies have proposed non-contact methods for monitoring respiration and measuring respiratory volume without relying on a spirometer [17,18,19,20,21,22]. The potential for non-contact respiratory volume measurement and diagnosis minimizes contact between medical staff and patients while offering the prospect of cost reduction.

This study proposes a decision support system for predicting COPD patients and non-patients through respiratory volume measurement. To predict patients and non-patients using respiratory volume, respiratory regions were identified, and respiratory data were accumulated. The accumulated respiratory data are output as the respiratory volume, average distance between expirations, average distance between inspirations, total respiratory rate, respiratory patterns, and visual representations such as respiratory volume graphs that capture overall trends and visualize expiration/inspiration tendencies. The accumulated respiratory data are converted into scores with assigned weights to classify COPD patients and non-patients.

The study’s key contributions are as follows:

- The respiratory characteristics and patterns were analyzed using thermal imaging camera-based respiratory recording. The respiratory data were analyzed using metrics such as the total respiratory volume, average distance between expirations, average distance between inspirations, and total respiratory rate. Additionally, graphs were generated to visually confirm respiratory patterns. The proposed method enables the identification of personalized respiratory characteristics.
- After applying Z-score scaling, scoring was performed using weighted summation, allowing for visual interpretation of the data. This approach demonstrated the potential to differentiate between COPD patients and the non-patient group.
- Through a study on the classification of COPD patients and the non-patient group using thermal imaging camera-based respiratory recordings, the potential of this method as an auxiliary tool for non-contact medical examination and diagnosis has been suggested.

## 2. Related Works

### 2.1. COPD

Chronic obstructive pulmonary disease (COPD) affects millions of individuals worldwide, with its prevalence steadily increasing [23]. The primary symptoms of COPD include coughing, sputum production, fatigue, and shortness of breath [24,25]. COPD impacts patients through reduced lung function and diminished quality of life (QoL) [26]. Among the major symptoms of COPD, shortness of breath arises due to the decline in lung function. This decline occurs gradually over an extended period, and patients often experience a cycle of adverse effects as they limit their activity range and duration to avoid the discomfort caused by shortness of breath during physical activities [27]. COPD is challenging to cure once it develops, but treatment is possible at any stage. The goals of treatment include preventing symptom progression, maintaining and improving quality of life, and reducing mortality rates [28]. Therefore, the early diagnosis of COPD plays a critical role. Currently, the standard diagnosis for COPD relies on lung function measurements using a spirometer [29]. Additionally, chest X-ray examinations are often performed to differentiate COPD patients from smokers and those with other respiratory diseases [30]. The Global Initiative for Chronic Obstructive Lung Disease (GOLD) guidelines [31] provide extensive discussions on various diagnostic methods for COPD. Diagnosis using spirometry primarily involves measuring the forced expiratory volume in one second (FEV₁) and the forced vital capacity (FVC). A patient is considered to have COPD when the ratio of these two values decreases to below 0.7 [25,31]. However, the current standard diagnostic method for COPD may yield inaccurate results depending on the skill level of the medical staff or the compliance of the patient. Moreover, it may lack the sensitivity required to accurately detect physiological changes in patients [32].

To enhance diagnostic accuracy and enable early detection, various methods for diagnosing COPD have recently been proposed [22,29,30]. In this study aimed to develop a COPD diagnostic support system by analyzing comfortable breathing, which is less affected by patient compliance. The research utilized four features—total respiratory volume, average distance between expirations, average distance between inspirations, and total respiratory rate—employing a Z-score-based weighted composite score to distinguish COPD.

### 2.2. Z-Score

The Z-score is a measure that represents how far a data point is from the mean in terms of standard deviation. It quantitatively evaluates the relative position of a specific value within a distribution and can detect anomalies based on deviations [33]. Additionally, scaling allows the normalization of columns from different categories, making it possible to sum or average the scores [34]. The advantage of the Z-score is that it simplifies data categories, facilitating comparison and computation. It also enables the identification of relative positions within the data and can be utilized in statistical analysis [35]. The Z-score formula is as shown in Equation (1). *Z* represents the result of applying the Z-score, where *X* is the data value, μ is the mean, and σ is the standard deviation.

(1) $$Z=\frac{\left(X-\mu \right)}{\sigma }\;\;\;\;\;\;\;\;\;\;\;\;\;\;\;\;\;\;\;\;\;\;\;\;\;\;\;\;\;$$

DeVore et al. [36] conducted a Z-score and percentile analysis of heart circumference data measured from the 4-chamber view of 144 fetuses between 20 and 35 weeks of gestation. The study proposed a systematic methodology for calculating the Z-scores and percentiles of fetal biometric indices and applied it to real data to present the analysis results. The results of this study suggest that Z-scores enable the analysis of how fetal heart circumference data correspond to specific age ranges and how far they deviate from the mean.

Yadav et al. [37] calculated the metabolic syndrome Z-score by summing the standardized values of cardio-metabolic risk factors. The study reported changes in the metabolic syndrome Z-score following a 12-week lifestyle intervention conducted on 54 adults with metabolic syndrome. The study proposed a method for standardizing metabolic syndrome into Z-scores and summing the values of each column. The 12-week changes were evaluated and presented as a single score dataset.

This study scaled heterogeneous data categories, including the total respiratory volume, average distance between expirations, average distance between inspirations, and total respiratory rate, using Z-scores. A threshold was determined based on the ROC curve, classifying values above the threshold as non-patients and those below as patients.

### 2.3. Medical Image Preprocessing Through Filtering

Noisy medical images act as obstacles to accurate disease diagnosis [38]. In particular, medical image preprocessing is considered an essential step in computer vision, with various algorithms proposed to remove noise and enhance image quality through preprocessing [39,40,41,42]. Sagheer et al. [38] introduces preprocessing methods for ultrasonography, MRI [43], CT [44,45], and PET [46] data, presenting filtering techniques tailored to the characteristics of each data type, including spatial domain filters, transform domain filters, statistical techniques, sparsity and low-rank-based techniques, and deep learning-based methods.

Spatial domain filtering is a method that removes noise by directly processing the pixel values of an input image. Techniques such as Gaussian averaging, mean, median, and bilateral filtering are included in spatial domain filters and are used to eliminate specific noise in medical images [38]. The primary goal of noise removal in medical imaging is to eliminate noise while preserving the target structures. Among these, smoothing is used to reduce image noise and remove unnecessary details to emphasize the target. Common smoothing filtering methods include mean filtering, median filtering, and Gaussian filtering.

The median filter [47] is a nonlinear smoothing technique that selects the median value from the pixel values surrounding a target pixel and assigns it to the target pixel. This filtering method replaces the target pixel’s value with the median value of its neighboring pixels. In the median filtering process, a k×k window (kernel) is centered on the target pixel, and all pixel values within this window are sorted in ascending order. The median value from this sorted sequence is then calculated, and the target pixel value is replaced with this median value. This process is repeated as the window moves across the entire image. The formula for the median filter is as shown in Equation (2).

(2) $$g\left(x,y\right)=Median\{f(s,t)|\left(s,t\right)\in W\}$$

$g\left(x,y\right)$ represents the pixel value in the filtered image, and $f\left(s,t\right)$ represents the pixel value in the original image.

The Gaussian filter [48] is a linear filter in which each pixel value is obtained as a weighted average of the pixel itself and its surrounding pixel values. Based on the Gaussian distribution, it performs smoothing by calculating the weighted average of the target pixel and its neighbors. After defining a k×k window (kernel) and standard deviation (σ), normalization is performed. The formula for the Gaussian filter is as shown in Equation (3).

(3) $$G\left(x,y\right)=\frac{1}{2\pi \sigma^{2}}e^{-\frac{x^{2}+y^{2}}{2\sigma^{2}}}$$

G(x,y) represents the value at a specific position in the Gaussian kernel, σ is the standard deviation of the Gaussian distribution, and x,y denote the distance from the center of the kernel.

Bilateral filtering [49,50] is a method that reduces noise while preserving edges through a nonlinear combination of pixel values within the kernel. It maintains the details of the image and preserves texture by considering color differences between pixels. This filter uses two parameters: spatial standard deviation ($\sigma_{s}$) and range standard deviation ($\sigma_{r}$), to perform image preprocessing. The formula for the bilateral filter is as shown in Equation (4).

(4) $$I^{\prime }\left(x,y\right)=\frac{1}{W}\sum_{(i,j)\in S}G_{s}(\|(x,y)-\left(i,j\right)\|)\cdot G_{r}(|I\left(x,y\right)-I\left(x,y\right)-I\left(i,j\right)|)\cdot I(i,j)$$

$I^{\prime }\left(x,y\right)$ represents the pixel value in the filtered result image, and $I\left(i,j\right)$ represents the pixel value in the original image. $G_{s}$ is the spatial Gaussian filter that calculates weights based on distance, and $G_{r}$ is the range Gaussian filter that calculates weights based on pixel value differences. W is the normalization constant, which is the sum of all weights. Finally, S represents the kernel area where the filter is applied.

Section 3.1 of this paper applied a median filter, Gaussian filter, and bilateral filter to reduce non-respiratory noise.

## 3. Methods

The purpose of thermal imaging-based respiratory pattern analysis is to distinguish between COPD patients and non-patients by identifying respiratory characteristics. This study was conducted in accordance with the Institutional Review Board (IRB No. 2023-10-012) conducted at Soonchunghyang University Cheonan Hospital. We record side-view thermal images of subjects breathing naturally in a relaxed state to monitor and document their expirations. The recording lasts approximately one minute, and the captured thermal images are segmented into image sequences, which are then fed into the pipeline for respiratory pattern analysis.

**Figure 1:**
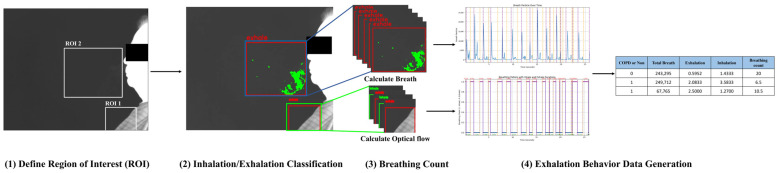
Overview of the proposed method.

The respiratory analysis process involves several steps: First, two regions of interest (ROIs) are designated in the initial frame—ROI 1 on the chest to differentiate between inspiration and expiration, and ROI 2 in front of the mouth to detect respiration. The optical flow is then calculated within ROI 1 to distinguish between inspiration and expiration. When ROI 1 indicates expiration, respiratory changes in ROI 2 are tracked by counting pixels that exceed a threshold difference between current and previous frames. This expiratory behavior is visualized as a respiratory waveform graph and quantified into features such as the total respiratory volume, average distance between expirations and inspirations, and total respiratory rate. The data processing pipeline is illustrated in Figure 1.

**Figure 2:**
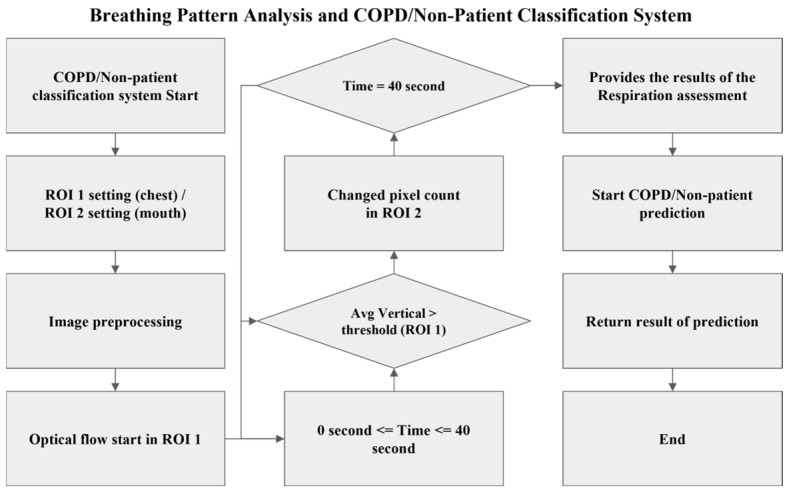
Flowchart for respiratory pattern and COPD/non-patient analysis.

The flowchart for respiratory pattern analysis and COPD patient and non-patient classification is shown in Figure 2. When initiating the classification of COPD patients and non-patients, ROI 1 and ROI 2 are first defined, followed by image preprocessing. The preprocessed image sequence undergoes analysis to distinguish inspiration and expiration using optical flow. If the video duration is less than 40 s, chest movements within the ROI 1 area are assessed. If an expiration is detected, respiratory data accumulation begins through pixel counting. Once the video duration reaches 40 s, the accumulated respiratory data are analyzed, and the results are presented. Based on the presented results, four columns are scaled and weighted for scoring, providing the classification outcome for COPD patients and non-patients. The four elements used in the respiratory data analysis are summarized in Table 1.

**Figure 3:**
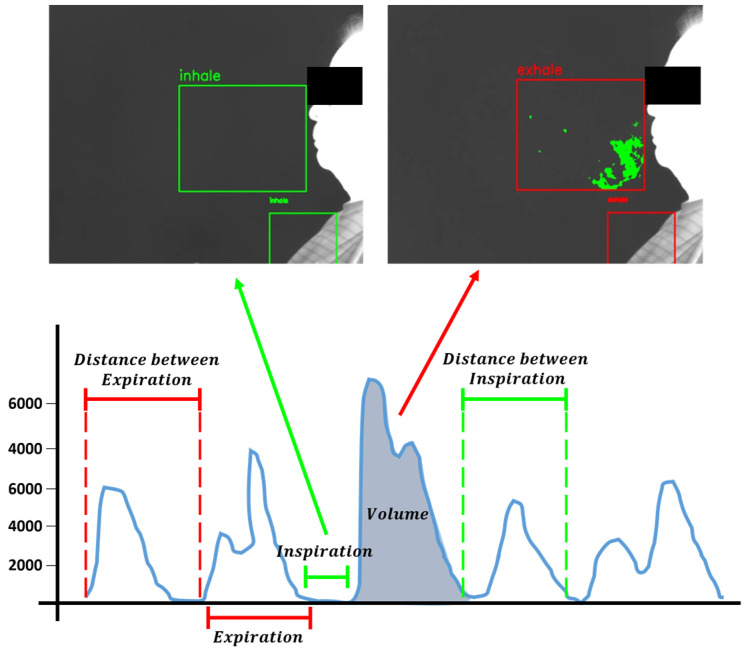
The measurement and differentiation methods for each element.

The total respiratory volume is the sum of the areas quantified across n expiratory phases. The average distance between expirations represents the average distance calculated by measuring the time from the start of one expiration to the start of the next expiration. Similarly, the average distance between inspirations represents the average distance calculated by measuring the time from the start of one inspiration to the start of the next inspiration. Finally, the total respiratory rate represents the frequency of respiratory cycles. The measurement methodology and the method used to distinguish each data element are illustrated in Figure 3.

### 3.1. Experimental Design and Data Collection

**Figure 4:**
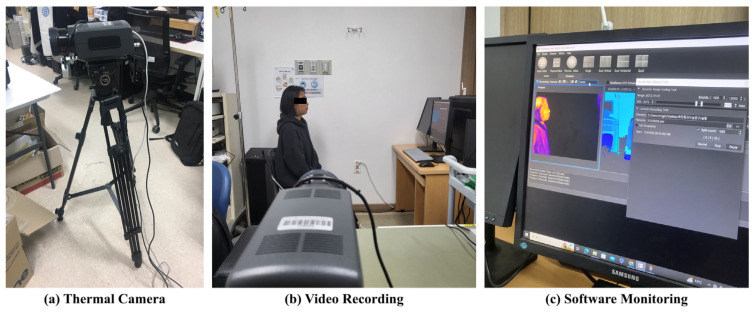
Experimental environment for video data collection. (**a**) Thermal imaging camera, (**b**) example of respiration posture, and (**c**) software.

Prior to data collection using a thermal camera, the experimental setup involved selecting an appropriate location and designing a method to ensure the acquisition of clean respiratory thermal image data. The thermal camera model used in this study was the FLIR-A6788sc (640 × 512 @30 Hz), Teledyne FLIR LLC, Wilsonville, OR, USA, capable of capturing not only the subject’s body temperature but also heat emissions and respiratory patterns. The custom software utilized for data acquisition was developed by a collaborating laboratory. Real-time monitoring of respiratory thermal images is possible, and the thermal images can be saved in BTI format. Additionally, the software provides a Dynamic Range Scaling Toolbar feature, allowing the conversion of thermal images into Grayscale, RGB, and other formats, enabling diverse methods for data analysis. The environment for data collection is illustrated in Figure 4. The data collection environment is shown in Table 2.

Data collection was conducted at Soonchunhyang University Cheonan Hospital. To distinguish between COPD patients and non-patients, individuals with a medical opinion indicating no relation to COPD were classified as the non-patient group, and video data were collected. The recordings were conducted in a sealed environment, and the indoor temperature was maintained at 18 °C to ensure accurate imaging of high-temperature gases, as required by the characteristics of the thermal camera. During video recording, only the experimenter and the subject were present in the laboratory to prevent disturbances in respiratory patterns caused by the movements of others. Each thermal image recording was conducted for one minute per individual. To capture natural breathing in a resting state, the subjects were seated and allowed to take sufficient rest before starting the recording. During data collection, movements other than breathing were minimized, and the subjects maintained a fixed gaze straight ahead. In this study, thermal image recordings were conducted for 20 individuals in the patient group and 20 individuals in the non-patient group, followed by analyses for each group. The participants involved in the experiment are shown in Table 3.

### 3.2. Determination of the Respiratory Area

In this study, two regions of interest (ROIs) were defined to reduce noise signals other than respiration and isolate the respiratory area. The first region (ROI 1) was set on the chest, while the second region (ROI 2) was positioned in front of the mouth with a size of 250 × 200  pixels. ROI 1 tracks chest movement to differentiate between inspiration and expiration. ROI 2 collects respiratory data when ROI 1 is determined to be in the expiratory state. The settings for each ROI were individually adjusted based on the subject’s body shape, the position of the nose and mouth, and differences in respiratory patterns. Accordingly, ROI 1 was placed on the area with the greatest movement during inspiration and expiration, while ROI 2 was positioned as close as possible to the nose and mouth.

### 3.3. Image Preprocessing

The collected video data were converted to grayscale. Unnormalized recordings, affected by factors such as indoor temperature, and the subject’s body temperature were manually normalized using the Dynamic Range Scaling Toolbar. Normalization was necessary because, despite setting the indoor temperature to 18 °C prior to recording, various environmental factors could cause temperature fluctuations. The normalized videos were converted to MP4 format, and image preprocessing was performed using the proposed method. The videos captured by the thermal camera contained a mixture of respiration signals and other noise. Even if the subject momentarily held their respiration just before recording, residual respiration signals remained in the air. Therefore, a preprocessing step is required to clearly distinguish respiration signals before respiration analysis. Preprocessing was performed using the median filter, Gaussian filter, and bilateral filter provided by OpenCV.

**Figure 5:**
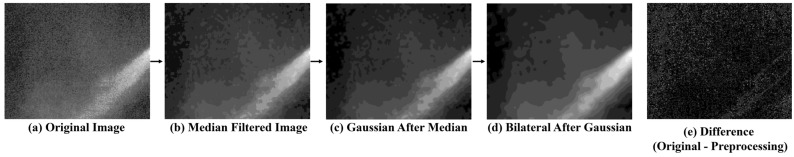
(**a**) Original image, (**b**) result of applying the median filter, (**c**) result of additional application of the Gaussian filter, (**d**) final result of applying the bilateral filter, and (**e**) comparing the original image with the preprocessed image.

First, the median filter and Gaussian filter were used to remove noise caused by salt-and-pepper noise and random points generated by ambient air. After removing extreme noise with the median filter, the Gaussian filter was applied to smooth the image and eliminate residual respiration noise. The kernel size for both the median filter and Gaussian filter is 5 × 5. Subsequently, the bilateral filter was used to remove residual noise while preserving texture. The kernel size for the bilateral filter is 9 × 9. The differences in preprocessing steps and the results of noise removal are shown in Figure 5. Starting from the original image on the left, filters are applied sequentially. The image on the far right (e) shows the result of applying all filters to the original image, highlighting the noise that has been removed.

**Figure 6:**
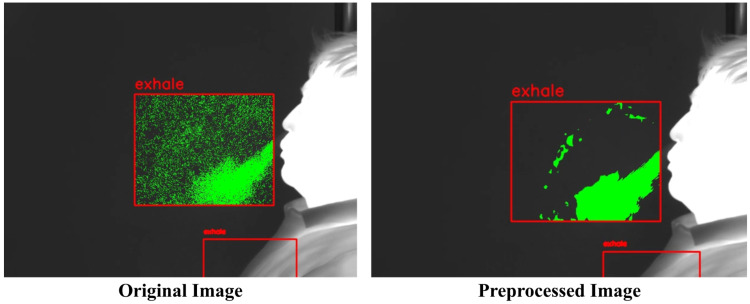
Comparison of respiratory visualization between original and preprocessed images.

The visual results are displayed in Figure 6. In the original video, noise unrelated to respiration was detected alongside respiratory movements due to pixel color changes during recording. However, in the preprocessed videos using the median filter, Gaussian filter, and bilateral filter, noise unrelated to respiration was significantly reduced, enabling more accurate identification of respiratory movements.

### 3.4. Analysis of Respiration Patterns

The preprocessed thermal images are input frame by frame for respiratory pattern analysis. Each image sequence, input on a per-frame basis, is classified as inspiration or expiration based on the results of optical flow calculated within ROI 1, set on the chest area. To minimize errors caused by minor chest vibrations or coughing, the optical flow threshold was set to 0.05. This excludes small vertical movements of the chest, such as those occurring when suppressing a cough, from the analysis. When the chest rises and inspiration is determined, respiration tracking and data collection in ROI 2 are only performed when ROI 1 is determined as expiration and are not conducted during inspiration.

To reduce noise other than respiration, the threshold was set to 1, so that pixel differences between the current and previous frames below 1 were not accumulated into the total respiratory volume of the corresponding frame. Respiratory patterns are analyzed for 40 s after the video starts. The reason for analyzing 40 s of respiration is that the respiration during this period was the most regular, and this approach minimizes issues such as interruptions in recording caused by irregular camera calibrations.

**Figure 7:**
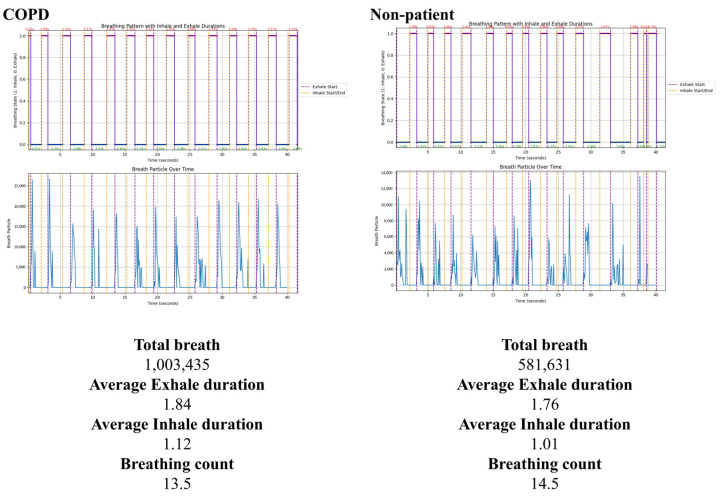
Analysis results of the participant’s respiratory pattern.

In ROI 1, chest movement is tracked along the y-axis using the optical flow algorithm. Expiration and inspiration are differentiated based on specific conditions, with a threshold applied to correct minor vibrations. When ROI 1 transitions to the expiration state, respiration is monitored from 0 to 40 s. In ROI 2, all pixels compare the color change between the current frame’s pixel and the previous frame’s pixel. If the color change is positive, the number of changed pixels is accumulated. When ROI 1 transitions to the inspiration state, all pixel values in ROI 2 are reset to zero. After verifying color changes for all pixels, the frame time is updated, and the number of pixels (total respiratory volume) per frame is recorded. Examples of the total respiratory volume graph and output data are shown in Figure 7. Based on y-axis movement, expiration is represented as 0 in the upward direction, while inspiration is represented as 1 in the downward direction. Additionally, the duration of each inspiration and expiration is annotated within the respective segments of the graph. The lower section of the graph illustrates the participant’s respiratory volume over time. Purple dashed lines indicate the start times of expirations, and yellow dashed lines indicate the start times of inspirations.

## 4. Data Analysis and Experiment, Results, and Discussion

This section explains the analysis and experiments conducted on respiratory analysis data, the results of the experiments, and the implications and limitations of this study. The data used for the analysis include the total respiratory volume, average distance between expirations, average distance between inspirations, and total respiratory rate. The experimental group consisted of 20 COPD patients and 20 non-patients as the control group. The COPD group consisted of individuals who were definitively diagnosed by a pulmonologist. The non-patients selected as the control group included “healthy adults” as well as individuals with a history of respiratory conditions or those currently visiting the hospital for the treatment of non-COPD respiratory diseases without a prior diagnosis of COPD. Table 4 shows the content related to respiratory diseases other than COPD in the control group of non-patients.

### 4.1. Analysis and Experiment

To analyze the data, respiratory characteristics were compared between 20 participants from the COPD group and non-patients. The comparison was conducted using the averages of each group to analyze the differences in tendencies. The comparison of characteristics between the patient group and the non-patient group for each measurement session is shown in Table 5.

#### 4.1.1. Data Analysis

**Figure 8:**
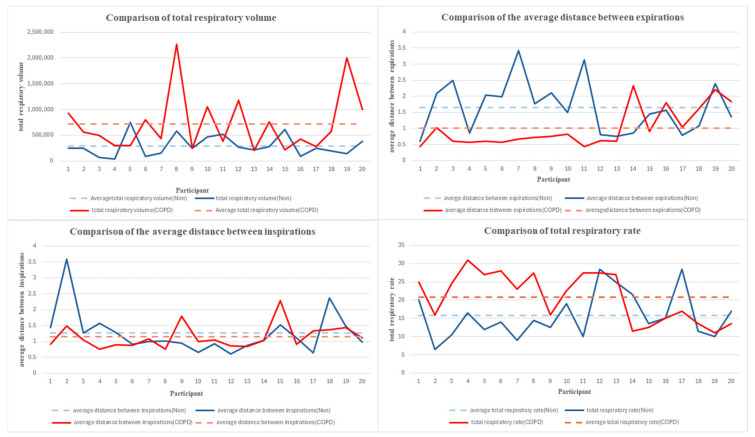
Comparison graph of measured features between the COPD group and non-patient group.

The total respiratory volume and total respiratory rate were higher in the COPD patient group, while the average distance between expirations and the average distance between inspirations were higher in the non-patient group. To analyze these differences individually, the four measurement items were visualized using graphs. Each graph is displayed in Figure 8. The dashed lines indicate the mean values for each group. The graphs marked in red represent the COPD group, while those in shades of blue represent the non-patient group.

In this study, the tendencies of COPD patient and non-patient groups were visually confirmed through graphical representations. However, simple linear classification methods posed potential for error. To overcome this limitation, a scoring method based on Z-score scaling was applied. This approach integrates scaled data while reflecting the interrelationships among the features of each column. Consequently, this methodological improvement enabled visual comparative analysis between the COPD patient and non-patient groups, facilitating more accurate classification. This approach is considered to contribute to improving the accuracy of COPD diagnosis.

#### 4.1.2. Experiment

This study distinguishes between COPD patients and non-patients using a scoring method that allows for visual comparison. All column data were scaled to a range with a mean of 0 and a standard deviation of 1 using Z-scores. Subsequently, simulations were performed with randomly assigned weights for each column to achieve integrated scoring. Weights were assigned to all four columns, each within a range of 1 to 50. A total of 1000 iterations of weight selection were performed for the simulation, and the weights that produced the best results were selected. The criteria for selecting the optimal weights are based on ensuring a balanced tendency to distinguish between COPD patients and non-patients, achieving a Youden’s index value exceeding 0.70, and prioritizing models with higher recall for COPD when accuracy is identical. The final selected weights are presented in Table 6.

The total respiratory volume exhibits a strong correlation with COPD (0.52) and an AUC value of 0.80, demonstrating excellent predictive capability as an independent variable. Based on these characteristics, it was assigned a high weight, designating it as a key contributing variable in the weighted composite score. The total respiratory rate, with a correlation of 0.34, shows a relatively lower association with COPD. However, its AUC value of 0.68 indicates a strong predictive capability, justifying the assignment of a moderate weight. The average distance between expirations has a negative correlation with COPD (−0.44), indicating a relatively strong association with COPD. However, its AUC value of 0.24 indicates a poor performance as an independent predictive variable. In contrast, the average distance between inspirations shows a minimal correlation with COPD (−0.06), but its AUC value of 0.46 highlights a better predictive capability as an independent variable.

**Figure 9:**
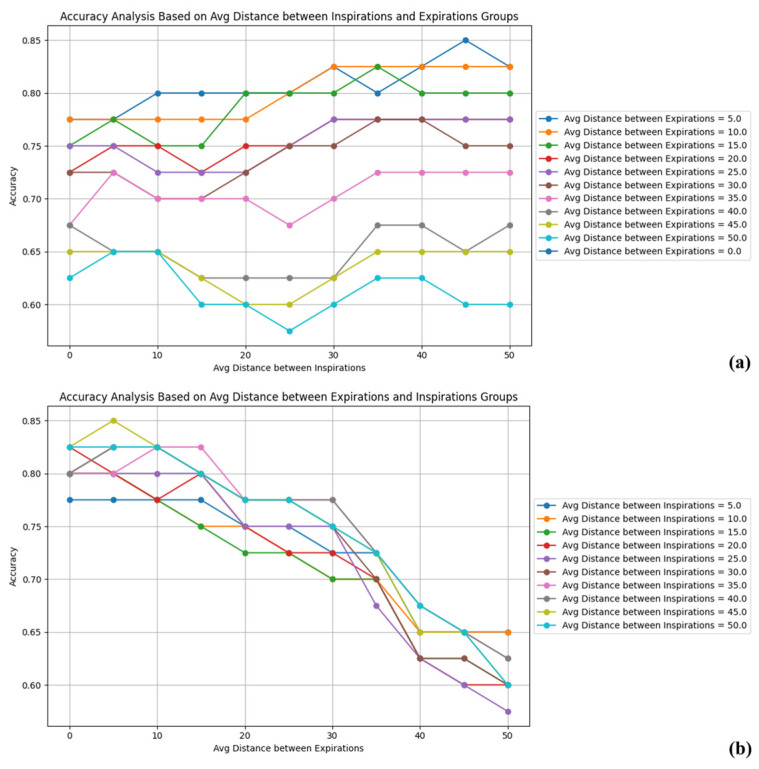
Analysis results of weight selection for average distance between expirations (**b**) and average distance between inspirations (**a**).

To validate the weight selection criteria for the average distance between expirations and the average distance between inspirations, an analysis was performed by comparing different weights. The results of the analysis are shown in Figure 9.

The analysis outcomes, as observed in Figure 9a, revealed that altering the weight of the average distance between expirations caused significant fluctuations in accuracy. In contrast, as shown in graph Figure 9b, changes in the weight of average distance between inspirations resulted in a relatively stable trend in accuracy. This experiment confirmed that setting a high weight for the average distance between expirations negatively impacts accuracy. Conversely, appropriately adjusting the weight of average distance between inspirations contributes to maintaining or improving accuracy. Based on these findings, the final weights for the average distance between expirations and average distance between inspirations were determined, taking into account their impact on accuracy. Prior to assigning weights, Z-score scaling was applied to all elements.

The Z-score-based scoring formula is provided in Equation (5).

(5) $$Score=(z_{TRV}\times w_{TRV})+(z_{ADE}\times w_{ADE})+(z_{ADI}\times w_{ADI})+(z_{TRR}\times w_{TRR})$$

$z_{TRV}$ represents the total respiratory volume, $z_{ADE}$ indicates the average distance between expirations, $z_{ADI}$ denotes the average distance between inspirations, and $z_{TRR}$ signifies the total respiratory rate. $w_{TRV}$, $w_{ADE}$, $w_{ADI}$, and $w_{TRR}$ refer to the weight assigned to each feature. To calculate the score, the assigned weight for each data point is multiplied by the respective feature value, and the results are summed to produce the final score.

The sequence for scoring-based classification of COPD patients and non-patients involves several steps. First, each of the four columns is scaled into a distribution with a mean of 0 and a standard deviation of 1 using Z-scores. These Z-score scaled columns are then multiplied by weights assigned based on simulation results. The four weighted columns are summed to generate a composite score. The optimal threshold is determined by selecting the value at the optimal point of the ROC curve, identified using Youden’s index. The formula for Youden’s index is as shown in Equation (6).

(6) $$\mathrm{Youde}n^{’}s\;\mathrm{Index}=TPR(False\;Positive\;Rate)-FPR(True\;Positive\;Rate)$$

The threshold value is finalized based on Youden’s index and accuracy. Each data point is classified as COPD if its score meets or exceeds the threshold, or as a non-patient if it falls below the threshold.

To validate the proposed model as a clinically meaningful diagnostic support tool, the minimal clinically important difference (MCID) [51] method was employed to compare the four features and the composite score. Based on the MCID criteria, improvements were observed in distinguishing between COPD patients and non-patients using the score-based approach. The AUC value of the weighted composite score was 0.84, and the comparison with the AUC-ROC values of each individual factor is shown in Table 7.

Consequently, the total respiratory rate, average distance between inspirations, and average distance between expirations demonstrated that the weighted composite score provides a significantly stronger predictive capability for distinguishing between COPD patients and non-patients compared to each individual factor. In contrast, the total respiratory volume showed a minimal difference in AUC compared to the weighted composite score and did not exceed the MCID threshold. This indicates that the total respiratory volume also contributes substantially to the prediction of COPD patients and non-patients. While the predictive power of the weighted composite score is relatively higher, total respiratory volume alone serves as an important predictive variable. The evaluation using the MCID method revealed that not all variables contribute equally to the prediction of COPD patients and non-patients through the weighted composite score. However, the combination of the three remaining variables, excluding the total respiratory volume, enhanced the predictive performance and suggests the potential to yield superior predictive outcomes. In contrast, the average distance between expirations exhibited a low AUC value when considered independently, indicating limited standalone predictive power. However, it was included in the analysis considering its complementary role through interactions with other variables during the analytical process. The detailed results of verification through MCID are shown in Table 7.

### 4.2. Result

In this study, data from a population of 40 individuals were used to distinguish between COPD patients and non-patients. The differentiation was performed using four elements extracted from video-based analysis. The detailed results for precision, recall, and F1-score are presented in Table 8. Notably, the high performance in the classification of COPD patients and non-patients is regarded as a significant factor in the medical field.

The proposed model demonstrates strength in predicting COPD, minimizing the likelihood of missing actual COPD (recall = 0.95). However, it may incorrectly predict some non-patients as COPD patients (recall = 0.75). However, both predictions of COPD and non-patient predictions achieve a recall of 0.75 or higher, allowing approximately 75% accuracy in distinguishing COPD patients and non-patients. This highlights the potential applicability of the proposed model in medical settings where detection of COPD is critical.

**Figure 10:**
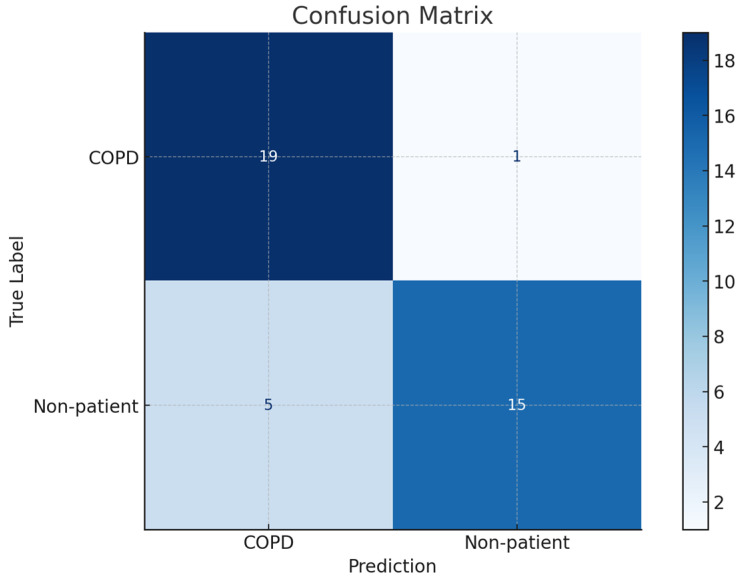
Confusion matrix for COPD and control group classification results.

The confusion matrix for the classification results of COPD patients and non-patients is shown in Figure 10. Out of 40 participants, the model accurately classified 19 out of 20 COPD patients as patients and correctly identified 15 out of 20 non-patients as non-patients. Based on these results, the calculated sensitivity was 0.95, and the specificity was 0.75, indicating that while the proposed model has a very high ability to accurately detect COPD, there exists a possibility of misclassifying non-patients as patients.

### 4.3. Discussion

This study approached respiratory pattern analysis and the classification of COPD patients and non-patients using a method distinct from the existing research and demonstrated the feasibility of distinguishing COPD patients and non-patients with a significant degree of accuracy. We attempted to distinguish between COPD patients and control subjects using a score-based approach through weighted summation of all four parameters. The parameters employed in the study include the total respiratory volume, total respiratory rate, average distance between inspirations, and average distance between expirations. Among these, three parameters—total respiratory volume (AUC = 0.80), total respiratory rate (AUC = 0.69), and average distance between inspirations (AUC = 0.46)—exhibited potential contributions to COPD and control subject prediction. However, the average distance between expirations showed a relatively limited predictive utility with an AUC of 0.24. The rationale for retaining the average distance between expirations parameter in this study is presented in Table 9.

The contributions of this study are as follows:

- The potential for personalized respiratory characteristic and pattern analysis through respiratory video recording

This study analyzed personalized respiratory patterns through respiratory videos captured with a thermal camera. The results demonstrate that recorded respiratory videos can provide quantitative data on an individual’s respiratory patterns, respiratory volume, expiration and inspiration patterns and durations, and respiratory rate. Furthermore, the analysis can extend to various expiratory activities (e.g., prolonged expiration or deep breathing) to identify detailed respiratory patterns. Additionally, future studies could potentially extend to include research on various pulmonary diseases such as apnea, asthma, and pneumonia.

- The potential for distinguishing between COPD patients and non-patients through respiration in a relaxed state

Unlike conventional pulmonary function measurement methods, this study explored the potential for distinguishing between COPD patients and non-patients through respiration in a relaxed state. The findings of this study suggest that differences in respiratory volume and patterns between COPD patients and non-patients may exist even in a relaxed state. This research highlights the potential for future studies to include patients with various respiratory disorders.

- The potential for application as a non-contact diagnostic support tool

This study highlights the potential for application as a non-contact diagnostic support tool, in contrast to traditional pulmonary function measurement methods that require contact-based approaches such as spirometers. It utilizes a method of capturing respiration through a thermal camera rather than attaching specific devices to the body or requiring oral contact. Therefore, with further research to enhance accuracy and robustness, it demonstrates potential for utilization as a non-contact tool for medical diagnosis and treatment support.

Therefore, this study is expected to expand into various research directions related to respiration in the future. However, the conclusions drawn from this study have certain limitations. These include the potential decrease in classification accuracy with the addition of more data and the inconvenience caused by setting ROC curve thresholds for additional data classification. The limitations of this study are as follows:

- Limitations due to the small sample size and data imbalance

The data collection for this study was conducted at Soonchunhyang University Cheonan Hospital. The thermal imaging camera used for data collection was a military-grade camera, which was only available during the co-researcher’s visits to Korea. As the same model was not available domestically, the data collection period faced certain limitations. Additionally, due to a medical strike, it was challenging to recruit a sufficient number of patients and non-patients visiting the hospital. Ultimately, the dataset used for COPD patient and non-patient classification in this study comprised 40 samples, including 20 patients and 20 non-patients. Due to the limited sample size of 40, there is a possibility that the classification criteria were derived based on a localized dataset, which may result in reduced accuracy when classifying new data.

Future research will focus on collecting additional data through collaboration with co-researchers to validate the generalizability of the proposed model. Additionally, efforts will be made to enhance predictive performance by employing various machine learning and deep learning algorithms.

- Limitations in the number of features used

In this study, the classification of COPD patients and non-patients was based on four features: total respiratory volume, average distance between expirations, average distance between inspirations, and total respiratory rate. Although these features were scaled and weighted according to their importance, the limited number of features may pose constraints on the depth of the analysis.

Future research will aim to address these limitations by developing a sophisticated model that incorporates the diverse physiological characteristics of patients and non-patients. This effort will focus on enhancing predictive performance to support the development of a more sophisticated diagnostic support system.

## 5. Conclusions

This study proposed a method for distinguishing between COPD patients and non-patients through preprocessing of thermal camera-recorded thermal images, respiratory pattern analysis, and refinement of respiration data.

To remove noise other than respiration and analyze accurate respiration data, two regions of interest (ROIs) were defined in the first frame of the thermal images: ROI 1, which tracks chest movements to distinguish between inspiration and expiration, and ROI 2, which covers the mouth and nose area where respiration occurs. The thermal recording was divided into image sequences and input frame by frame. Each image was preprocessed using the median filter, Gaussian filter, and bilateral filter to reduce noise and isolate respiration, followed by respiratory pattern analysis. When ROI 1 transitioned to the “expiration” state, respiration data were accumulated in ROI 2. The accumulation method involved calculating the difference between the current frame and the previous frame within ROI 2 and counting the number of pixels with positive differences on a frame-by-frame basis. Respiration data accumulation was performed from 0 to 40 s, considering that the recording typically starts just before expiration and accounting for the camera’s characteristics, including uncertainties in interruption cycles caused by calibration. The accumulation process was terminated when the thermal recording reached 40 s. Subsequently, the results included respiratory patterns, graphs illustrating expiration and inspiration patterns, total respiratory volume, average distance between expirations, average distance between inspirations, and total respiratory rate.

The respiratory information used to distinguish between COPD patients and non-patients was selected based on four parameters: total respiratory volume, average distance between expirations, average distance between inspirations, and total respiratory rate. The respiratory data were scaled using Z-scores, and the scaled data were assigned weights based on simulation results, then summed to generate a score. Based on the analysis of each score, a threshold derived from the value of the Receiver Operating Characteristic (ROC) curve was selected to distinguish between COPD patients and non-patients. Values below the threshold were classified as non-patients, while values at or above the threshold were classified as COPD. Using the proposed method, an 85% classification accuracy was achieved in distinguishing between COPD patients and non-patients.

This study performed the classification of inspiration and expiration and the calculation of the total respiratory volume, average distance between expirations, average distance between inspirations, and total respiratory rate, as well as the distinction between individuals with COPD and non-COPD individuals based on thermal imaging videos. Through this research, various respiratory information, including overall video-based respiratory patterns, respiratory volume, expiration distance, inspiration distance, and respiratory rate, could be identified. Additionally, the study demonstrated the potential for COPD classification based on respiratory information.

Future research will aim to overcome the limitations in the number of data samples and features in this study to enhance the accuracy of COPD patient and non-patient classification. To achieve this, additional features will be collected, including the physiological characteristics of each subject (e.g., gender, weight, height, and age) and key COPD risk factors such as smoking status. The study will employ data augmentation algorithms like SMOTE to validate usability and integrate the augmented datasets into machine-learning analyses. Furthermore, research will focus on precise respiratory tracking using U-Net-based models to advance the mapping and detection of respiratory volume during expiration by leveraging videos of respiratory regions.

## Figures and Tables

**Table 1:** Results of respiration data analysis.

Respiration Data			
Total Respiratory Volume	Average Distance between Expirations	Average Distance between Inspirations	Total Respiratory Rate

**Table 2:** Computer performance and software.

Software/Parameters	Value
Windows 10	64-bit
Programming Language	Python 3.8.10
CPU	Intel Core i7-9700 CPU @ 3.00 GHZ
GPU	NVIDIA GeForce RTX 2080
RAM	32 GB

**Table 3:** Participant information.

Type of Dataset	Number of Participants	Average Age
COPD	20	72.9
Non-patient	20	48.2

**Table 4:** Respiratory disease status in non-patient groups.

Control Subjects (Non-Patients)	
Healthy adults	14
Non-COPD pulmonary disorders (Emphysema, tuberculosis, bronchiectasis, bronchitis, pulmonary embolism, etc.)	6

**Table 5:** Average respiratory data of COPD patients and non-patients.

	Total Respiratory Volume	Avg Distance Between Expirations	Avg Distance Between Inspirations	Total Respiratory Rate
Non-patient	292,177	1.652137	1.260358	15.750000
COPD	720,140	1.007284	1.141885	20.825000

**Table 6:** Correlation analysis results and assigned weights.

	Total Respiratory Volume	Avg Distance Between Expirations	Avg Distance Between Inspirations	Total Respiratory Rate
Correlation Analysis				
COPD	0.52	−0.44	−0.06	0.34
Elements AUC				
AUC-ROC	0.80	0.24	0.46	0.69
Column Weight				
Weight	48.0	3.0	46.0	33.0

**Table 7:** Results of validation through MCID.

	Total Respiratory Volume	Total Respiratory Rate	Avg Distance Between Inspirations	Avg Distance Between Expirations
AUC	0.80	0.69	0.46	0.24
Observed Difference	0.37	0.14	0.37	0.59
MCID Threshold (Upper)	0.08	0.11	0.13	0.12
MCID Met	False	True	True	True

**Table 8:** Interpretation of classification results for COPD patients and non-patients.

	Precision	Recall	F1-Score
COPD (Positive Class)	0.79	0.95	0.86
Non-patient (Negative Class)	0.94	0.75	0.83

**Table 9:** Result comparison by number of factors.

		Precision	Recall	F1-Score	Sensitivity	Specificity
Four factors employed	COPD (Positive Class)	0.79	0.95	0.86	0.95	0.75
	Non-patient (Negative Class)	0.94	0.75	0.83		
Three factors employed	COPD (Positive Class)	0.78	0.9	0.837	0.9	0.75
	Non-patient (Negative Class)	0.882	0.75	0.81		

## Data Availability

The data presented in this study are available on request from the corresponding author.
